# Supplementary material for: Deep learning models for RNA secondary structure prediction (probably) do not generalize across families
Source: Bioinformatics. 2022 Jun 24;38(16):3892–9. doi: 10.1093/bioinformatics/btac415 (PMC9364374; doi:10.1093/bioinformatics/btac415)
Supplement: btac415_Supplementary_Data [file btac415_supplementary_data.pdf]

Supplementary Information

Deep learning models for RNA secondary structure prediction (probably) do not generalise across families

Preprint, compiled June 9, 2022

Marcell Szikszai [1,*], **Michael Wise** [1,2], **Amitava Datta** [1], **Max Ward** [1,3], **and David H. Mathews** [4]

[1]Department of Computer Science & Software Engineering, The University of Western Australia, Perth, WA, Australia
[2]The Marshall Centre for Infectious Diseases Research and Training, The University of Western Australia, Perth, WA, Australia
[3]Department of Molecular and Cellular Biology, Harvard University, Cambridge, MA, USA
[4]Department of Biochemistry & Biophysics, Center for RNA Biology, and Department of Biostatistics & Computational Biology, University of Rochester, Rochester, NY, USA

Contents

1 Demonstrative model grid-search **2**

1.1 *k*-fold . . . 2

1.2 Family-fold . . . 3

2 Demonstrative model AUC vs. Epoch **4**

2.1 *k*-fold . . . 4

2.2 Family-fold . . . 4

3 Training reproducibility **5**

3.1 RNA-state-inference . . . 5

3.2 CROSS . . . 5

3.3 DMfold . . . 5

3.4 SPOT-RNA . . . 5

3.5 E2Efold . . . 5

3.6 RPRes . . . 5

3.7 MXfold2 . . . 5

3.8 UFold . . . 5

4 Mean minimum tree edit distance **6**

4.1 Method . . . 6

4.2 Results . . . 6

5 Demonstrative model results **7**

6 $\Delta\Delta G'$ nudges **8**

***correspondence:** marcell.szikszai@research.uwa.edu.au

1 Demonstrative model grid-search

1.1 k-fold

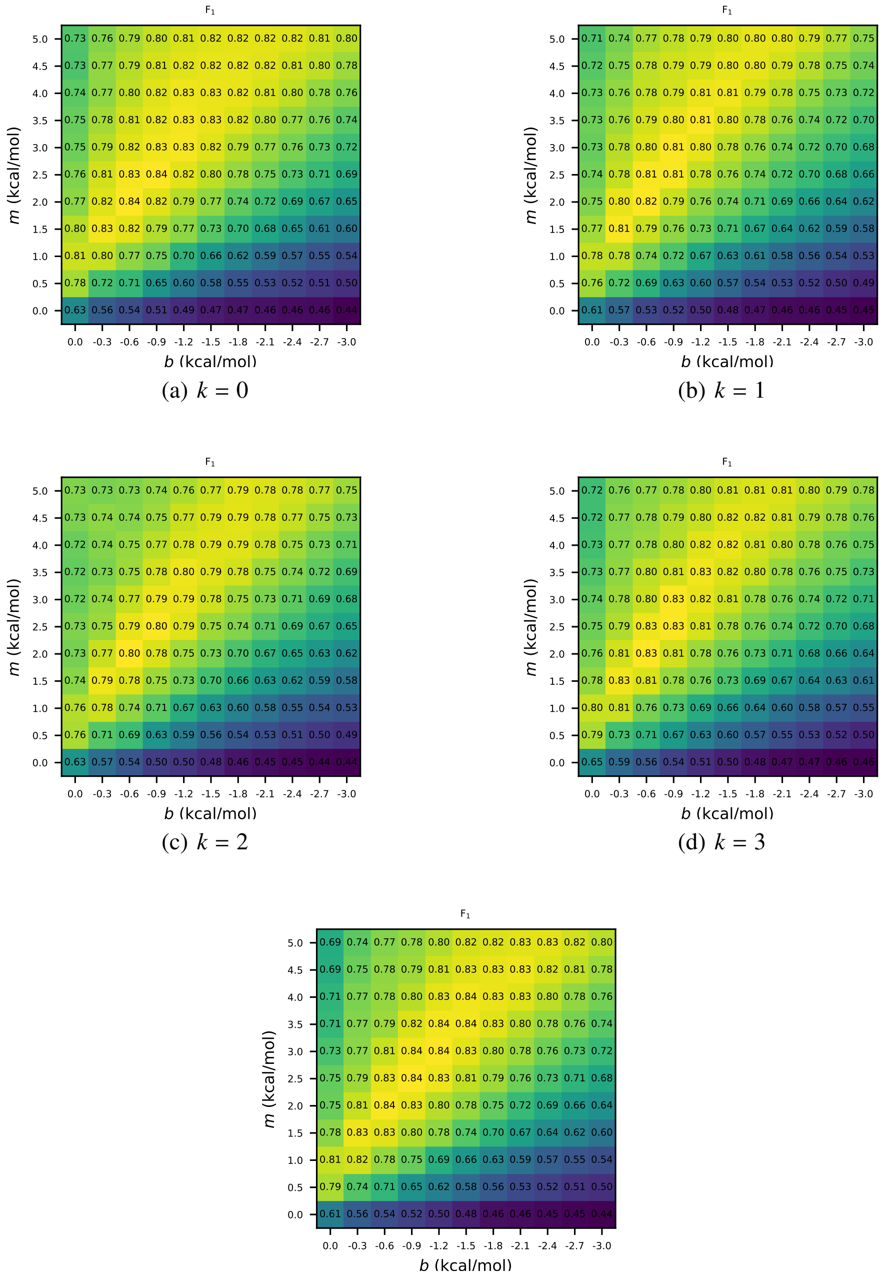

(a) $k = 0$ (b) $k = 1$

(c) $k = 2$ (d) $k = 3$

(e) $k = 4$

Figure 1: Results of the slope m and intercept b grid-search for the pseudo-free energy Equation 1 free parameters for k-fold cross-validation, broken down by k.

1.2 Family-fold

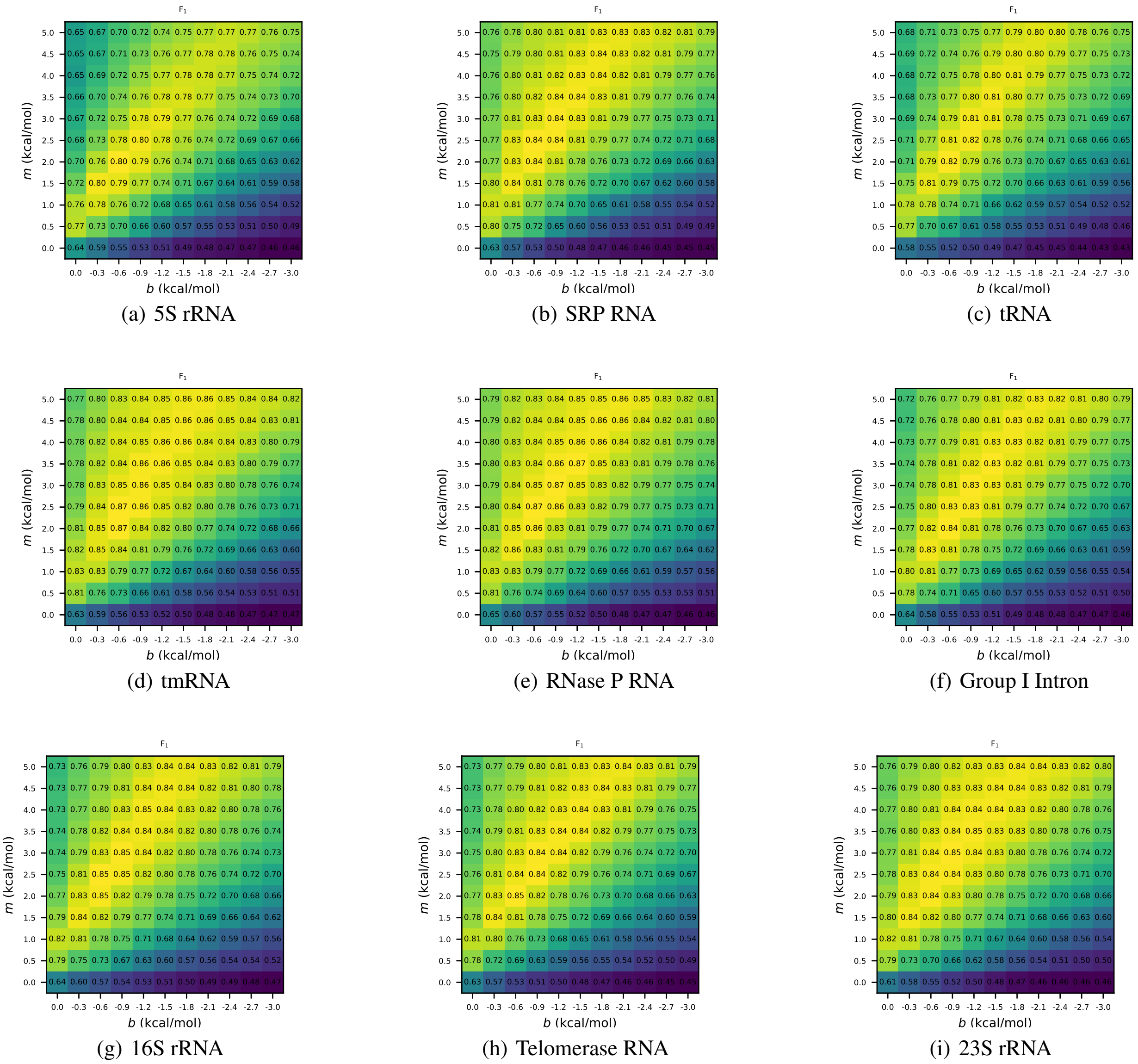

(a) 5S rRNA (b) SRP RNA (c) tRNA (d) tmRNA (e) RNase P RNA (f) Group I Intron (g) 16S rRNA (h) Telomerase RNA (i) 23S rRNA

Figure 2: Results of the slope m and intercept b grid-search for the pseudo-free energy Equation 1 free parameters for family-fold cross-validation, broken down by family.

2 Demonstrative model AUC vs. Epoch

2.1 *k-fold*

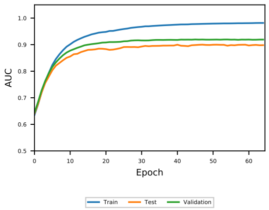

(a) $k = 0$

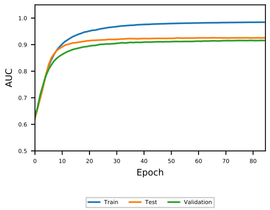

(b) $k = 1$

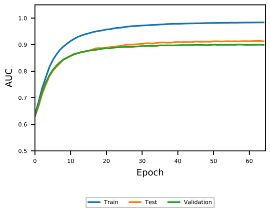

(c) $k = 2$

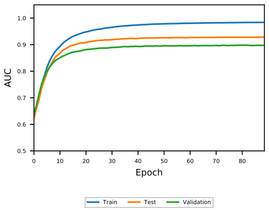

(d) $k = 3$

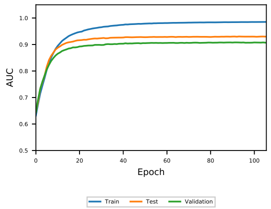

(e) $k = 4$

Figure 3: Performance of *k*-fold training, validation, and testing sets on our demonstrative model vs. training epoch.

2.2 *Family-fold*

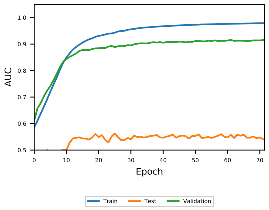

(a) 5S rRNA

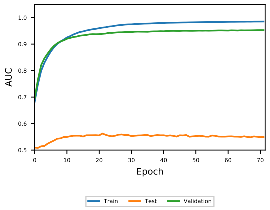

(b) SRP RNA

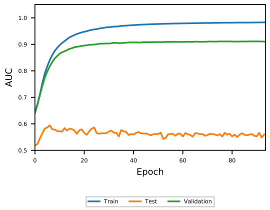

(c) tRNA

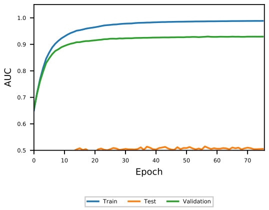

(d) tmRNA

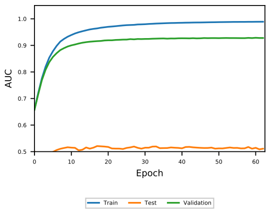

(e) RNase P RNA

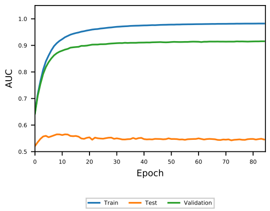

(f) Group I Intron

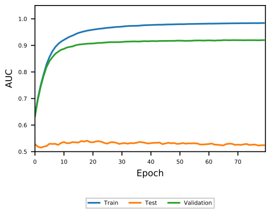

(g) 16S rRNA

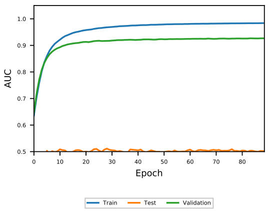

(h) Telomerase RNA

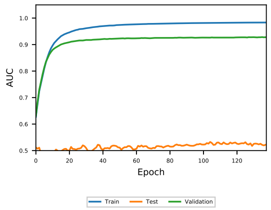

(i) 23S rRNA

Figure 4: Performance of family-fold training, validation, and testing sets on our demonstrative model vs. training epoch.

3 Training reproducibility

3.1 *RNA-state-inference*

Source code is published, however, since inter-family performance is addressed in their work [1], we did not feel it was necessary to benchmark on our dataset.

3.2 *CROSS*

No source code or training methods are provided for CROSS, and as such, we were unable to re-train their model on our dataset.

3.3 *DMfold*

The authors of DMfold publish the entire source code on Github, however, we were unable to re-train their network. The published Git repository contains bugs that make it impossible to reproduce their methods, or meaningfully debug their code. To attempt to run the program, we used Python 3.5.2 and Tensorflow 1.5.0 as specified in the provided Software Guide.

We have opened an issue on Github[1] detailing the problems we ran into trying to re-train their model, but received no response from the authors.

3.4 *SPOT-RNA*

No source code or training methods are provided for SPOT-RNA, and as such, we were unable to re-train their model on our dataset.

3.5 *E2Efold*

Source code is published, and training is reproducible, although the memory requirements are high (176GB+).

We contacted the authors via email (sent 2021-08-10), and they were helpful in assisting with re-training their method. However, as the MXfold2 [2] and UFold [3] papers have already benchmarked E2Efold for inter-family performance, we did not feel it was necessary to benchmark on our dataset.

3.6 *RPRes*

The authors of RPRes do publish the entire source code on Github, however, we were unable to re-train their network as per their instructions. Attempting to run RPRes out of the box throws a `RuntimeError: DataLoader worker exited unexpectedly` exception. There are very limited instructions provided on how to run the code, and no `requirements.txt` or other environment details provided.

We have attempted to contact the corresponding authors via email (sent 2021-06-04) for assistance running their code, but received no response at the time of writing.

3.7 *MXfold2*

Source code is published, and training is reproducible.

3.8 *UFold*

The authors of UFold publish the entire source code on Github, however, we were unable to re-train their network as per their instructions out of the box. After opening an issue on Github[2], the authors were helpful in fixing some issues with the training code. After the authors' help, we were able to reproduce their training methodology, albeit without the use of synthetic sequences [3] for pre-training.

[1] https://github.com/linyuwangPHD/RNA-Secondary-Structure-Database/issues/3
[2] https://github.com/uci-cbcl/UFold/issues/4

4 Mean minimum tree edit distance

4.1 Method

Pair-wise tree-editing distances [4] were calculated using ViennaRNA's [5] RNAdistance[3] program between all sequences in the training and testing sets. For each sequence in the testing set, the minimum tree-editing distance to the sequences in the training set was found. These minimums were then averaged across each cross-validation fold.

4.2 Results

Table 1: Average minimum tree-editing distances of *k*-fold cross-validation training and testing sets. Note the lower distance (i.e. higher structural similarity) compared to family-fold cross-validation sets from Table 2.

Test set	Train #	Test #	Distance
0-fold	2783	773	16.51
1-fold	2783	773	14.68
2-fold	2783	773	15.17
3-fold	2783	773	16.29
4-fold	2783	773	14.92
Mean			15.51

Table 2: Average minimum tree-editing distances of *k*-fold cross-validation training and testing sets. Note the higher distance (i.e. lower structural similarity) compared to *k*-fold cross-validation sets from Table 1.

Test set	Train #	Test #	Distance
5s-fold	2324	1283	54.50
16s-fold	3419	67	150.03
23s-fold	3465	15	175.60
grp1-fold	3412	74	182.08
RNaseP-fold	3070	454	161.65
srp-fold	2653	918	99.01
telomerase-fold	3447	35	185.46
tmRNA-fold	3063	462	168.42
tRNA-fold	2978	557	38.13
Mean			134.99

[3] https://www.tbi.univie.ac.at/RNA/RNAdistance.1.html

5 DEMONSTRATIVE MODEL RESULTS

Table 3: Detailed results of family-fold cross-validation on our demonstrative model.

		PPV		Sensitivity		F_1		
**Family**	**#**	**Baseline**	**Model**	**Baseline**	**Model**	**Baseline**	**Model**	**p-value**[4]
16S rRNA	67	0.54	0.47	0.62	0.50	0.58	0.48	<0.001
23S rRNA	15	0.70	0.53	0.77	0.56	0.73	0.54	0.033
5S rRNA	1283	0.61	0.50	0.66	0.44	0.63	0.46	<0.001
RNase P RNA	454	0.53	0.48	0.58	0.49	0.55	0.48	<0.001
Group I Intron	74	0.50	0.47	0.57	0.52	0.53	0.49	0.002
SRP RNA	918	0.62	0.51	0.67	0.51	0.64	0.50	<0.001
tRNA	557	0.79	0.67	0.82	0.64	0.80	0.65	<0.001
Telomerase RNA	35	0.43	0.39	0.60	0.53	0.50	0.45	0.005
tmRNA	462	0.41	0.39	0.46	0.42	0.43	0.41	<0.001
Total	3865							
Mean		0.57	0.49	0.64	0.51	0.60	0.50	<0.001

Table 4: Detailed results of *k*-fold cross-validation on our demonstrative model.

		PPV		Sensitivity		F_1		
**Family**	**#**	**Baseline**	**Model**	**Baseline**	**Model**	**Baseline**	**Model**	**p-value**[4]
16S rRNA	67	0.54	0.58	0.62	0.61	0.58	0.60	0.482
23S rRNA	15	0.70	0.66	0.77	0.70	0.73	0.68	0.468
5S rRNA	1283	0.61	0.93	0.66	0.96	0.63	0.94	<0.001
RNase P RNA	454	0.53	0.65	0.58	0.67	0.55	0.66	<0.001
Group I Intron	74	0.50	0.51	0.57	0.55	0.53	0.53	0.784
SRP RNA	918	0.62	0.80	0.67	0.83	0.64	0.81	<0.001
tRNA	557	0.79	0.97	0.82	0.98	0.80	0.97	<0.001
Telomerase RNA	35	0.43	0.55	0.60	0.69	0.50	0.61	0.001
tmRNA	462	0.41	0.65	0.46	0.63	0.43	0.64	<0.001
Total	3865							
Mean		0.57	0.70	0.64	0.74	0.60	0.72	<0.001

Table 5: Area Under the Receiver Operating Characteristic Curve (AUC) for *k*-fold cross-validation and family-fold cross validation.

		AUC		
**Family**	**#**	***k*-fold**	**family-fold**	**p-value**
16S rRNA	67	0.77	0.72	0.009
23S rRNA	15	0.79	0.73	0.080
5S rRNA	1283	0.95	0.72	<0.001
RNase P RNA	454	0.81	0.71	<0.001
Group I Intron	74	0.73	0.72	0.387
SRP RNA	918	0.88	0.73	<0.001
tRNA	557	0.97	0.79	<0.001
Telomerase RNA	35	0.76	0.68	<0.001
tmRNA	462	0.82	0.68	<0.001
Total	3865			
Mean		0.83	0.72	<0.001

[4] Calculated from F_1 scores.

6 $\Delta\Delta G'$ NUDGES

Table 6: F_1 score of negative $\Delta\Delta G'$ nudges by family. Red indicates degraded performance, while green indicates improved performance over baseline predictions. Values with * indicate statistically significant results (Table 8).

$\Delta\Delta G$	16S rRNA	23S rRNA	5S rRNA	RNase P RNA	Group I Intron RNA	SRP RNA	tRNA	Telomerase RNA	tmRNA
-1.00	0.49*	0.57*	0.51*	0.48*	0.46*	0.55*	0.64*	0.28*	0.32*
-0.98	0.49*	0.58*	0.51*	0.48*	0.46*	0.55*	0.64*	0.28*	0.33*
-0.96	0.49*	0.58*	0.52*	0.49*	0.46*	0.55*	0.64*	0.29*	0.33*
-0.94	0.49*	0.58	0.52*	0.49*	0.47*	0.55*	0.65*	0.29*	0.33*
-0.92	0.49*	0.58	0.52*	0.49*	0.47*	0.55*	0.65*	0.29*	0.33*
-0.90	0.49*	0.58	0.52*	0.49*	0.47*	0.56*	0.65*	0.29*	0.33*
-0.88	0.49*	0.58	0.52*	0.49*	0.47*	0.56*	0.65*	0.29*	0.33*
-0.86	0.49*	0.59	0.52*	0.49*	0.47*	0.56*	0.66*	0.30*	0.33*
-0.84	0.49*	0.60	0.52*	0.49*	0.47*	0.56*	0.66*	0.32*	0.33*
-0.82	0.49*	0.60	0.52*	0.49*	0.47*	0.56*	0.66*	0.32*	0.33*
-0.80	0.50*	0.60	0.52*	0.50*	0.47*	0.56*	0.66*	0.32*	0.33*
-0.78	0.50*	0.60	0.53*	0.50*	0.48*	0.56*	0.67*	0.32*	0.33*
-0.76	0.50*	0.62	0.53*	0.50*	0.48*	0.56*	0.67*	0.32*	0.33*
-0.74	0.50*	0.62	0.53*	0.50*	0.48*	0.57*	0.67*	0.33*	0.34*
-0.72	0.50*	0.62	0.53*	0.50*	0.48*	0.57*	0.68*	0.33*	0.34*
-0.70	0.51*	0.62	0.53*	0.50*	0.48*	0.57*	0.68*	0.33*	0.34*
-0.68	0.51*	0.62	0.53*	0.50*	0.48*	0.57*	0.68*	0.33*	0.34*
-0.66	0.51*	0.62	0.53*	0.50*	0.49*	0.57*	0.68*	0.33*	0.34*
-0.64	0.51*	0.63	0.54*	0.51*	0.49*	0.57*	0.69*	0.34*	0.34*
-0.62	0.52*	0.63	0.54*	0.51*	0.49*	0.58*	0.69*	0.34*	0.35*
-0.60	0.52*	0.63	0.54*	0.51*	0.49*	0.58*	0.69*	0.34*	0.35*
-0.58	0.52*	0.63	0.54*	0.51*	0.49*	0.58*	0.70*	0.35*	0.35*
-0.56	0.52*	0.63	0.54*	0.51*	0.49*	0.58*	0.70*	0.35*	0.35*
-0.54	0.52*	0.63	0.54*	0.51*	0.49*	0.58*	0.70*	0.37*	0.36*
-0.52	0.52*	0.63	0.54*	0.51*	0.49*	0.58*	0.70*	0.37*	0.36*
-0.50	0.52*	0.64	0.54*	0.51*	0.49*	0.59*	0.71*	0.37*	0.36*
-0.48	0.53*	0.65	0.55*	0.52*	0.49*	0.59*	0.71*	0.37*	0.36*
-0.46	0.53*	0.65	0.55*	0.52*	0.49*	0.59*	0.71*	0.38*	0.37*
-0.44	0.53*	0.67	0.55*	0.52*	0.49*	0.59*	0.72*	0.38*	0.37*
-0.42	0.53*	0.67	0.55*	0.52*	0.50*	0.59*	0.73*	0.38*	0.37*
-0.40	0.53*	0.68	0.56*	0.52*	0.50*	0.59*	0.73*	0.39*	0.37*
-0.38	0.53*	0.68	0.56*	0.52*	0.50*	0.59*	0.74*	0.39*	0.37*
-0.36	0.53*	0.69	0.56*	0.52*	0.50*	0.60*	0.74*	0.41*	0.38*
-0.34	0.54*	0.68	0.56*	0.52*	0.50*	0.60*	0.74*	0.41*	0.38*
-0.32	0.54*	0.71	0.57*	0.52*	0.50*	0.60*	0.74*	0.42*	0.38*
-0.30	0.55	0.73	0.57*	0.53*	0.50*	0.60*	0.74*	0.42*	0.38*
-0.28	0.55	0.73	0.57*	0.53*	0.50*	0.61*	0.75*	0.43*	0.39*
-0.26	0.55	0.73	0.57*	0.53*	0.51*	0.61*	0.75*	0.44*	0.39*
-0.24	0.56	0.73	0.58*	0.53*	0.51*	0.61*	0.75*	0.45*	0.39*
-0.22	0.56	0.73	0.58*	0.53*	0.51*	0.62*	0.76*	0.45*	0.40*
-0.20	0.56	0.73	0.58*	0.53*	0.51*	0.62*	0.76*	0.46	0.40*
-0.18	0.56	0.73	0.59*	0.54*	0.51*	0.62*	0.77*	0.47	0.40*
-0.16	0.56	0.72	0.59*	0.54*	0.52	0.62*	0.77*	0.48	0.40*
-0.14	0.56	0.72	0.60*	0.54*	0.52	0.63	0.78*	0.47	0.41*
-0.12	0.56	0.72	0.60*	0.54*	0.52	0.64	0.78*	0.47*	0.41*
-0.10	0.56	0.74	0.61*	0.54*	0.53	0.64	0.78*	0.47*	0.41*
-0.08	0.56	0.74	0.61*	0.54*	0.52	0.64	0.78*	0.48	0.42*
-0.06	0.57	0.74	0.62*	0.55	0.52*	0.64	0.79*	0.49	0.42*
-0.04	0.56	0.72	0.62*	0.55	0.53	0.64	0.80*	0.49	0.42*
-0.02	0.56	0.72	0.63*	0.55	0.53	0.63	0.80	0.49	0.43*
0.0	0.57	0.73	0.63	0.55	0.53	0.63	0.80	0.50	0.43

Table 7: F_1 score of positive $\Delta\Delta G'$ nudges by family. Red indicates degraded performance, while green indicates improved performance over baseline predictions. Values with * indicate statistically significant results (Table 9).

$\Delta\Delta G$	16S rRNA	23S rRNA	5S rRNA	RNase P RNA	Group I Intron RNA	SRP RNA	tRNA	Telomerase RNA	tmRNA
0.02	0.57	0.73	0.63	0.55	0.54	0.63	0.81	0.50	0.44*
0.04	0.57	0.72	0.63	0.55	0.53	0.63	0.81*	0.51	0.44*
0.06	0.56*	0.66	0.65*	0.55	0.53	0.63	0.80	0.52	0.45*
0.08	0.56	0.66	0.65*	0.56	0.53	0.62*	0.80	0.53	0.45*
0.10	0.56	0.68	0.65*	0.55	0.53	0.61*	0.80	0.53	0.46*
0.12	0.56	0.67	0.65*	0.55	0.54	0.60*	0.81	0.53	0.46*
0.14	0.55	0.67	0.64*	0.55	0.54	0.60*	0.81	0.53	0.46*
0.16	0.54*	0.68	0.64	0.54*	0.53	0.59*	0.80	0.52	0.46*
0.18	0.53*	0.66	0.62	0.54*	0.53	0.58*	0.79	0.52	0.46*
0.20	0.54*	0.66	0.61*	0.53*	0.53	0.56*	0.78*	0.51	0.46*
0.22	0.52*	0.67	0.60*	0.53*	0.49*	0.55*	0.76*	0.51	0.45*
0.24	0.50*	0.66	0.58*	0.52*	0.47*	0.54*	0.76*	0.49	0.44
0.26	0.49*	0.65	0.56*	0.50*	0.46*	0.51*	0.74*	0.47	0.44
0.28	0.48*	0.63	0.53*	0.49*	0.44*	0.47*	0.73*	0.45	0.43
0.30	0.46*	0.57*	0.51*	0.47*	0.41*	0.44*	0.70*	0.42*	0.43
0.32	0.45*	0.54*	0.47*	0.46*	0.38*	0.40*	0.67*	0.42*	0.41*
0.34	0.44*	0.50*	0.44*	0.44*	0.36*	0.36*	0.62*	0.41*	0.39*
0.36	0.40*	0.47*	0.41*	0.43*	0.32*	0.34*	0.58*	0.38*	0.38*
0.38	0.37*	0.45*	0.36*	0.41*	0.28*	0.31*	0.54*	0.36*	0.34*
0.40	0.34*	0.41*	0.32*	0.38*	0.25*	0.27*	0.49*	0.33*	0.31*
0.42	0.32*	0.33*	0.28*	0.36*	0.23*	0.25*	0.44*	0.30*	0.29*
0.44	0.29*	0.30*	0.24*	0.33*	0.21*	0.19*	0.41*	0.26*	0.25*
0.46	0.29*	0.25*	0.21*	0.30*	0.19*	0.16*	0.34*	0.23*	0.22*
0.48	0.27*	0.22*	0.16*	0.27*	0.15*	0.13*	0.26*	0.21*	0.20*
0.50	0.22*	0.21*	0.13*	0.24*	0.12*	0.10*	0.22*	0.20*	0.16*
0.52	0.19*	0.17*	0.10*	0.22*	0.10*	0.09*	0.17*	0.17*	0.13*
0.54	0.16*	0.15*	0.07*	0.19*	0.08*	0.08*	0.12*	0.16*	0.11*
0.56	0.15*	0.10*	0.05*	0.17*	0.07*	0.07*	0.10*	0.13*	0.10*
0.58	0.15*	0.09*	0.04*	0.15*	0.06*	0.06*	0.08*	0.12*	0.08*
0.60	0.11*	0.07*	0.03*	0.11*	0.04*	0.05*	0.06*	0.06*	0.06*
0.62	0.09*	0.06*	0.02*	0.09*	0.03*	0.04*	0.04*	0.05*	0.05*
0.64	0.06*	0.05*	0.02*	0.07*	0.03*	0.02*	0.04*	0.03*	0.04*
0.66	0.06*	0.05*	0.01*	0.06*	0.01*	0.02*	0.03*	0.03*	0.04*
0.68	0.05*	0.03*	0.01*	0.05*	0.01*	0.02*	0.03*	0.02*	0.03*
0.70	0.05*	0.03*	0.01*	0.04*	0.01*	0.01*	0.02*	0.02*	0.02*
0.72	0.05*	0.03*	0.01*	0.03*	0.01*	0.01*	0.02*	0.01*	0.02*
0.74	0.05*	0.01*	0.01*	0.03*	0.01*	0.01*	0.02*	0.01*	0.02*
0.76	0.05*	0.01*	0.01*	0.02*	0.01*	0.01*	0.02*	0.01*	0.02*
0.78	0.05*	0.01*	0.01*	0.02*	0.01*	0.01*	0.02*	0.01*	0.02*
0.80	0.05*	0.01*	0.01*	0.02*	0.01*	0.01*	0.02*	0.01*	0.02*
0.82	0.05*	0.01*	0.01*	0.02*	0.01*	0.01*	0.02*	0.01*	0.02*
0.84	0.05*	0.01*	0.01*	0.02*	0.01*	0.01*	0.02*	0.02*	0.02*
0.86	0.05*	0.01*	0.01*	0.02*	0.01*	0.01*	0.02*	0.02*	0.02*
0.88	0.04*	0.00*	0.01*	0.02*	0.01*	0.01*	0.02*	0.02*	0.02*
0.90	0.04*	0.00*	0.01*	0.02*	0.01*	0.01*	0.02*	0.02*	0.02*
0.92	0.04*	0.00*	0.01*	0.02*	0.01*	0.01*	0.02*	0.02*	0.02*
0.94	0.03*	0.00*	0.01*	0.02*	0.01*	0.01*	0.02*	0.01*	0.02*
0.96	0.03*	0.00*	0.01*	0.02*	0.01*	0.01*	0.02*	0.02*	0.02*
0.98	0.03*	0.00*	0.01*	0.02*	0.01*	0.01*	0.02*	0.02*	0.02*
1.00	0.03*	0.00*	0.01*	0.02*	0.01*	0.01*	0.02*	0.02*	0.02*
0.0	0.57	0.73	0.63	0.55	0.53	0.63	0.80	0.50	0.43

Table 8: Two-tailed paired t-tests for positive $\Delta\Delta G'$ nudges.

$\Delta\Delta G$	16S rRNA	23S rRNA	5S rRNA	RNase P RNA	Group I Intron RNA	SRP RNA	tRNA	Telomerase RNA	tmRNA
-1.00	<0.001	0.036	<0.001	<0.001	<0.001	<0.001	<0.001	<0.001	<0.001
-0.98	<0.001	0.037	<0.001	<0.001	<0.001	<0.001	<0.001	<0.001	<0.001
-0.96	<0.001	0.037	<0.001	<0.001	<0.001	<0.001	<0.001	<0.001	<0.001
-0.94	<0.001	0.051	<0.001	<0.001	<0.001	<0.001	<0.001	<0.001	<0.001
-0.92	<0.001	0.051	<0.001	<0.001	<0.001	<0.001	<0.001	<0.001	<0.001
-0.90	<0.001	0.052	<0.001	<0.001	<0.001	<0.001	<0.001	<0.001	<0.001
-0.88	<0.001	0.052	<0.001	<0.001	0.001	<0.001	<0.001	<0.001	<0.001
-0.86	<0.001	0.062	<0.001	<0.001	0.001	<0.001	<0.001	<0.001	<0.001
-0.84	<0.001	0.055	<0.001	<0.001	<0.001	<0.001	<0.001	<0.001	<0.001
-0.82	<0.001	0.055	<0.001	<0.001	<0.001	<0.001	<0.001	<0.001	<0.001
-0.80	<0.001	0.055	<0.001	<0.001	<0.001	<0.001	<0.001	<0.001	<0.001
-0.78	<0.001	0.055	<0.001	<0.001	0.001	<0.001	<0.001	<0.001	<0.001
-0.76	<0.001	0.076	<0.001	<0.001	<0.001	<0.001	<0.001	<0.001	<0.001
-0.74	<0.001	0.076	<0.001	<0.001	0.001	<0.001	<0.001	<0.001	<0.001
-0.72	<0.001	0.083	<0.001	<0.001	0.001	<0.001	<0.001	<0.001	<0.001
-0.70	0.001	0.083	<0.001	<0.001	<0.001	<0.001	<0.001	<0.001	<0.001
-0.68	0.001	0.083	<0.001	<0.001	<0.001	<0.001	<0.001	<0.001	<0.001
-0.66	0.001	0.085	<0.001	<0.001	0.001	<0.001	<0.001	<0.001	<0.001
-0.64	0.002	0.097	<0.001	<0.001	0.001	<0.001	<0.001	<0.001	<0.001
-0.62	0.002	0.103	<0.001	<0.001	0.001	<0.001	<0.001	<0.001	<0.001
-0.60	0.002	0.103	<0.001	<0.001	0.001	<0.001	<0.001	<0.001	<0.001
-0.58	0.004	0.099	<0.001	<0.001	0.001	<0.001	<0.001	<0.001	<0.001
-0.56	0.005	0.099	<0.001	<0.001	0.001	<0.001	<0.001	<0.001	<0.001
-0.54	0.005	0.089	<0.001	<0.001	0.001	<0.001	<0.001	<0.001	<0.001
-0.52	0.005	0.090	<0.001	<0.001	0.001	<0.001	<0.001	<0.001	<0.001
-0.50	0.006	0.129	<0.001	<0.001	0.001	<0.001	<0.001	<0.001	<0.001
-0.48	0.011	0.134	<0.001	<0.001	0.001	<0.001	<0.001	<0.001	<0.001
-0.46	0.014	0.132	<0.001	<0.001	0.001	<0.001	<0.001	<0.001	<0.001
-0.44	0.013	0.214	<0.001	<0.001	0.002	<0.001	<0.001	<0.001	<0.001
-0.42	0.010	0.214	<0.001	<0.001	0.003	<0.001	<0.001	<0.001	<0.001
-0.40	0.012	0.330	<0.001	<0.001	0.004	<0.001	<0.001	0.001	<0.001
-0.38	0.018	0.336	<0.001	<0.001	0.006	<0.001	<0.001	0.001	<0.001
-0.36	0.018	0.377	<0.001	<0.001	0.006	<0.001	<0.001	0.002	<0.001
-0.34	0.032	0.369	<0.001	<0.001	0.006	<0.001	<0.001	0.002	<0.001
-0.32	0.032	0.615	<0.001	<0.001	0.006	<0.001	<0.001	0.004	<0.001
-0.03	0.057	0.935	<0.001	<0.001	0.005	<0.001	<0.001	0.005	<0.001
-0.28	0.057	0.935	<0.001	<0.001	0.008	<0.001	<0.001	0.010	<0.001
-0.26	0.058	0.935	<0.001	<0.001	0.012	<0.001	<0.001	0.016	<0.001
-0.24	0.155	0.935	<0.001	<0.001	0.034	<0.001	<0.001	0.042	<0.001
-0.22	0.189	0.938	<0.001	<0.001	0.014	<0.001	<0.001	0.043	<0.001
-0.20	0.209	0.943	<0.001	<0.001	0.019	<0.001	<0.001	0.065	<0.001
-0.18	0.201	0.909	<0.001	<0.001	0.042	<0.001	<0.001	0.141	<0.001
-0.16	0.244	0.687	<0.001	0.003	0.080	0.002	<0.001	0.224	<0.001
-0.14	0.186	0.809	<0.001	0.008	0.099	0.275	<0.001	0.133	<0.001
-0.12	0.284	0.775	<0.001	0.008	0.080	0.394	<0.001	0.019	<0.001
-0.10	0.217	0.815	<0.001	0.001	0.211	0.430	<0.001	0.014	<0.001
-0.08	0.245	0.801	<0.001	0.007	0.117	0.347	<0.001	0.178	<0.001
-0.06	0.624	0.709	<0.001	0.318	0.039	0.095	0.017	0.279	<0.001
-0.04	0.303	0.243	0.004	0.321	0.317	0.092	0.046	0.200	<0.001
-0.02	0.209	0.308	0.006	0.749	0.491	0.654	0.054	0.233	<0.001

Table 9: Two-tailed paired t-tests for negative $\Delta\Delta G'$ nudges.

$\Delta\Delta G$	16S rRNA	23S rRNA	5S rRNA	RNase P RNA	Group I Intron RNA	SRP RNA	tRNA	Telomerase RNA	tmRNA
0.02	0.548	0.102	0.193	0.363	0.122	0.669	0.143	0.900	0.009
0.04	0.146	0.432	0.571	0.828	0.406	0.684	0.034	0.423	0.001
0.06	0.042	0.213	<0.001	0.652	0.276	0.181	0.643	0.318	<0.001
0.08	0.088	0.213	<0.001	0.408	0.383	0.007	0.733	0.121	<0.001
0.10	0.134	0.372	<0.001	0.825	0.631	<0.001	0.626	0.113	<0.001
0.12	0.117	0.327	0.001	0.651	0.864	<0.001	0.323	0.105	<0.001
0.14	0.074	0.345	0.013	0.181	0.697	<0.001	0.467	0.164	<0.001
0.16	0.044	0.379	0.350	0.046	0.812	<0.001	0.983	0.312	<0.001
0.18	0.011	0.269	0.121	0.016	0.512	<0.001	0.090	0.301	<0.001
0.20	0.031	0.257	<0.001	0.001	0.783	<0.001	0.025	0.603	<0.001
0.22	0.006	0.401	<0.001	<0.001	0.013	<0.001	<0.001	0.762	0.002
0.24	0.001	0.292	<0.001	<0.001	0.001	<0.001	<0.001	0.824	0.070
0.26	<0.001	0.198	<0.001	<0.001	<0.001	<0.001	<0.001	0.229	0.432
0.28	<0.001	0.124	<0.001	<0.001	<0.001	<0.001	<0.001	0.083	0.888
0.30	<0.001	0.011	<0.001	<0.001	<0.001	<0.001	<0.001	0.012	0.354
0.32	<0.001	0.004	<0.001	<0.001	<0.001	<0.001	<0.001	0.012	0.019
0.34	<0.001	0.002	<0.001	<0.001	<0.001	<0.001	<0.001	0.004	<0.001
0.36	<0.001	0.001	<0.001	<0.001	<0.001	<0.001	<0.001	0.001	<0.001
0.38	<0.001	0.001	<0.001	<0.001	<0.001	<0.001	<0.001	<0.001	<0.001
0.40	<0.001	<0.001	<0.001	<0.001	<0.001	<0.001	<0.001	<0.001	<0.001
0.42	<0.001	<0.001	<0.001	<0.001	<0.001	<0.001	<0.001	<0.001	<0.001
0.44	<0.001	<0.001	<0.001	<0.001	<0.001	<0.001	<0.001	<0.001	<0.001
0.46	<0.001	<0.001	<0.001	<0.001	<0.001	<0.001	<0.001	<0.001	<0.001
0.48	<0.001	<0.001	<0.001	<0.001	<0.001	<0.001	<0.001	<0.001	<0.001
0.50	<0.001	<0.001	<0.001	<0.001	<0.001	<0.001	<0.001	<0.001	<0.001
0.52	<0.001	<0.001	<0.001	<0.001	<0.001	<0.001	<0.001	<0.001	<0.001
0.54	<0.001	<0.001	<0.001	<0.001	<0.001	<0.001	<0.001	<0.001	<0.001
0.56	<0.001	<0.001	<0.001	<0.001	<0.001	<0.001	<0.001	<0.001	<0.001
0.58	<0.001	<0.001	<0.001	<0.001	<0.001	<0.001	<0.001	<0.001	<0.001
0.60	<0.001	<0.001	<0.001	<0.001	<0.001	<0.001	<0.001	<0.001	<0.001
0.62	<0.001	<0.001	<0.001	<0.001	<0.001	<0.001	<0.001	<0.001	<0.001
0.64	<0.001	<0.001	<0.001	<0.001	<0.001	<0.001	<0.001	<0.001	<0.001
0.66	<0.001	<0.001	<0.001	<0.001	<0.001	<0.001	<0.001	<0.001	<0.001
0.68	<0.001	<0.001	<0.001	<0.001	<0.001	<0.001	<0.001	<0.001	<0.001
0.70	<0.001	<0.001	<0.001	<0.001	<0.001	<0.001	<0.001	<0.001	<0.001
0.72	<0.001	<0.001	<0.001	<0.001	<0.001	<0.001	<0.001	<0.001	<0.001
0.74	<0.001	<0.001	<0.001	<0.001	<0.001	<0.001	<0.001	<0.001	<0.001
0.76	<0.001	<0.001	<0.001	<0.001	<0.001	<0.001	<0.001	<0.001	<0.001
0.78	<0.001	<0.001	<0.001	<0.001	<0.001	<0.001	<0.001	<0.001	<0.001
0.80	<0.001	<0.001	<0.001	<0.001	<0.001	<0.001	<0.001	<0.001	<0.001
0.82	<0.001	<0.001	<0.001	<0.001	<0.001	<0.001	<0.001	<0.001	<0.001
0.84	<0.001	<0.001	<0.001	<0.001	<0.001	<0.001	<0.001	<0.001	<0.001
0.86	<0.001	<0.001	<0.001	<0.001	<0.001	<0.001	<0.001	<0.001	<0.001
0.88	<0.001	<0.001	<0.001	<0.001	<0.001	<0.001	<0.001	<0.001	<0.001
0.90	<0.001	<0.001	<0.001	<0.001	<0.001	<0.001	<0.001	<0.001	<0.001
0.92	<0.001	<0.001	<0.001	<0.001	<0.001	<0.001	<0.001	<0.001	<0.001
0.94	<0.001	<0.001	<0.001	<0.001	<0.001	<0.001	<0.001	<0.001	<0.001
0.96	<0.001	<0.001	<0.001	<0.001	<0.001	<0.001	<0.001	<0.001	<0.001
0.98	<0.001	<0.001	<0.001	<0.001	<0.001	<0.001	<0.001	<0.001	<0.001
1.00	<0.001	<0.001	<0.001	<0.001	<0.001	<0.001	<0.001	<0.001	<0.001

REFERENCES

[1] Devin Willmott, David Murrugarra, and Qiang Ye. Improving RNA secondary structure prediction via state inference with deep recurrent neural networks. *Computational and Mathematical Biophysics*, 8(1):36–50, January 2020. ISSN 2544-7297. doi: 10.1515/cmb-2020-0002.

[2] Kengo Sato, Manato Akiyama, and Yasubumi Sakakibara. RNA secondary structure prediction using deep learning with thermodynamic integration. *Nature Communications*, 12(1):941, February 2021. ISSN 2041-1723. doi: 10.1038/s41467-021-21194-4.

[3] Laiyi Fu, Yingxin Cao, Jie Wu, *et al.* UFold: Fast and accurate RNA secondary structure prediction with deep learning. *Nucleic Acids Research*, page gkab1074, November 2021. ISSN 0305-1048. doi: 10.1093/nar/gkab1074.

[4] Bruce A. Shapiro and Kaizhong Zhang. Comparing multiple RNA secondary structures using tree comparisons. *Bioinformatics*, 6(4):309–318, October 1990. ISSN 1367-4803. doi: 10.1093/bioinformatics/6.4.309.

[5] Ronny Lorenz, Stephan H. Bernhart, Christian Höner zu Siederdissen, *et al.* ViennaRNA Package 2.0. *Algorithms for Molecular Biology*, 6(1):26, November 2011. ISSN 1748-7188. doi: 10.1186/1748-7188-6-26.
